# Development and validation of an interpretable machine learning model for acute radiation dermatitis in breast cancer

**DOI:** 10.3389/fonc.2025.1663293

**Published:** 2025-10-17

**Authors:** Xuejuan Duan, Yadong Liu, Yuguang Shang, Xiaomeng Lu, Yanhong Zhou, Liguo Liu, Zhikun Liu

**Affiliations:** ^1^ Department of Radiation Oncology, Fourth Hospital of Hebei Medical University, Shijiazhuang, Hebei, China; ^2^ Department of Oncology, Hebei General Hospital, Shijiazhuang, Hebei, China; ^3^ Second Department of Hepatopancreatobiliary Surgery, China-Japan Friendship Hospital, Beijing, China

**Keywords:** radiation dermatitis, predictive model, breast cancer, SHAP, radiotherapy

## Abstract

**Background and Purpose:**

Radiation dermatitis (RD), a common adverse reaction in breast cancer radiotherapy, impairs quality of life and increases healthcare burdens. Developing an effective risk prediction model is crucial for early high-risk patient identification and preventive interventions.

**Materials and Methods:**

This study enrolled 691 breast cancer patients undergoing postoperative radiotherapy at our center from February 1 to December 19, 2024. RD severity and correlates were monitored during and 2 weeks after radiotherapy. The dataset was divided into training (n=552) and test (n=139) cohorts. Fourteen machine learning algorithms were evaluated via 10-fold cross-validation, with model selection based on Area Under the Curve (AUC) and other metrics. Model reliability was verified using an internal hold-out test set, and SHAP analysis ensured interpretability.

**Results:**

Among 691 patients,52.68% (n=364) developed grade ≥2 acute RD. The random forest model performed best, achieving an AUC of 0.84 (95% CI: 0.807–0.873) in training and 0.748 (0.665–0.831) in testing, with training/testing sensitivity/specificity of 0.811/0.747 and 0.877/0.576, respectively. Calibration curves confirmed prediction-observation consistency. Decision curve analysis indicated 0.2–0.4 higher net benefits than “treat-all” or “treat-none” strategies at 25%–75% treatment thresholds. Shapley Additive exPlanations (SHAP) analysis identified Clinical Target Volume-Supraclavicular (CTVsc), Clinical Target Volume-Internal Mammary (CTVim), TNM stage II, and diabetic status as key predictors.

**Conclusion:**

This explainable machine learning model demonstrates robust discriminative power and clinical utility. Interpretability analysis revealed feature nonlinearities, providing a theoretical basis for personalized radiotherapy planning to reduce severe RD risk.

## 1 Introduction

Breast cancer remains the most prevalent malignancy among women, with its incidence and mortality rates continuing to rise rapidly in recent years, underscoring its substantial disease burden (1). As a critical component of multidisciplinary breast cancer treatment, radiotherapy effectively reduces local recurrence rates but concurrently induces RD, a key complication impeding therapeutic progress. Clinical evidence indicates that acute radiation dermatitis (ARD) occurs in 90-95% of radiotherapy patients, over half of the patients developed grade 2 or higher radiation dermatitis (1), significantly compromising quality of life and potentially leading to treatment discontinuation with adverse prognostic implications. Consequently, developing an accurate predictive model for severe RD could substantially enhance clinicians’ ability to identify high-risk patients and mitigate ARD incidence.

The rapid advancement of artificial intelligence has demonstrated machine learning’s potential in predicting radiotherapy-related toxicities. Existing studies have achieved partial progress: radiomics combined with dosiomics features reportedly improved the prediction of Grade ≥2 RD (AUC = 0.82) (2), while integrated multi-omics models identified clinical factors (smoking history, radiation dose) and serum cytokines (IL-4, IL-17) as robust predictors (3). Cui et al. developed a nomogram incorporating Body Mass Index (BMI), metabolic disorders (dyslipidemia, diabetes), and tumor characteristics (regional lymph node metastasis, proliferation index). However, the fact that only breast-conserving surgery patients were included limits its clinical generalizability. Additionally, the nomogram prediction model has insufficient ability to fit the non-linear relationships of high-dimensional data, leading to inadequate accuracy (4). Yuxiu Xie’s prospective study identified pre-radiotherapy BMI, diabetes, smoking history, elevated ferritin, hs-CRP, and CD3+ T-cell levels as independent risk factors for severe ARD. However, this study still uses the nomogram method and adopts the National Cancer Institute Common Terminology Criteria for Adverse Events (CTCAE), and does not include radiotherapy-related data (5). The limitations of the above studies collectively constrain model generalizability and clinical utility. This study aims to develop and validate a machine learning model to predict ≥ Grade 2 radiation dermatitis using clinical, treatment, and socioeconomic variables.

To bridge critical gaps in existing radiation dermatitis prediction models, this study establishes a comprehensive methodological framework integrating three synergistic innovations: a multidimensional predictor system synthesizing clinical, radiotherapeutic, and sociodemographic determinants; rigorously implemented Machine learning with systematic algorithmic validation; and SHAP-based interpretability safeguards ensuring coherence of machine learning model. Collectively, these advances deliver a robust and clinically actionable platform for personalized risk stratification. The findings revealed radiotherapy target parameters (CTVsc, CTVim), TNM Stage II, and diabetic status as critical drivers of severe radiation dermatitis (RD), significantly enhancing predictive accuracy for post-radiotherapy skin toxicity risk in breast cancer patients. This framework provides actionable decision support for clinical practitioners.

## 2 Methods

### 2.1 Study design and patient characteristics

This retrospective analysis evaluated breast cancer patients receiving adjuvant radiotherapy at our institution from February 1 to December 19, 2024. The follow-up ended on February 25, 2025. All participants provided written informed consent, ensuring ethical compliance. Inclusion criteria required: 1) age ≥18 years; 2) prior breast-conserving surgery or total mastectomy; 3) completion of whole-breast radiotherapy or chest wall ± regional nodal irradiation to ensure cohort homogeneity. Exclusion criteria encompassed: 1) previous radiotherapy history; 2) concurrent or prior malignancies; 3) concomitant chemotherapy; 4) pre-existing dermatological or active connective tissue disorders, minimizing confounding factors.

Radiotherapy commenced 4–6 weeks post-surgery or final chemotherapy cycle. All patients underwent Image-Guided Intensity-Modulated Radiation Therapy (IGRT-IMRT) with target volumes and organs-at-risk delineated per Radiation Therapy Oncology Group (RTOG) guidelines. Prescription doses followed: Breast-conserving surgery: Whole breast 43.5 Gy in 15 fractions or 50 Gy in 25 fractions, with tumor bed boost to 49.5 Gy/15fx or 60 Gy/25fx. Mastectomy: Chest wall and partial lymphatic drainage areas 45–50 Gy in 25 fractions, supplemented to 60–66 Gy to supraclavicular/internal mammary nodal metastases. All patients who have undergone mastectomy will use a bolus with a thickness of 0.5 cm during radiotherapy.

### 2.2 Variables and outcomes of interest

Combined with previous literature studies (1–5) and considering the universality of model application, we selected 31 clinically accessible predictors. For the missing values in the data, considering that our data satisfies the mechanism of missing at random, we adopted the method of data imputation, and subsequently used the imputed dataset for analysis. For numerical variables, the KNN imputation method was used, and for categorical variables, the mode imputation method was adopted. After excluding datasets with a data missing rate of >5% and those with high collinearity, this study collected demographic, treatment-related, and clinical outcome data (radiation dermatitis) from breast cancer patients. The dataset encompassed 21 variables: 13 clinical factors (age, height, weight, BMI, body surface area, diabetes status), 2 socioeconomic factors (education level, income tier), and 6 radiotherapy parameters (**Supplementary Material Table S1**). Variables were categorized as follows: Surgical approach: Breast-conserving surgery (BCS), Total mastectomy, Breast reconstruction. Tumor characteristics: Laterality (Left/Right/Bilateral), Pathological staging (AJCC 8th edition TNM), Histological type [Ductal Carcinoma *In Situ* (DCIS), Lobular Carcinoma *In Situ* (LCIS), Invasive breast carcinoma (IBC)]. Radiotherapy specifications: Target volume [Clinical Target Volume-Chest Wall (CTVcw), Clinical Target Volume-Breast Cancer (CTVbc), Clinical Target Volume-Supraclavicular (CTVsc), Clinical Target Volume-Internal Mammary (CTVim), Clinical Target Volume-Axilla (CTVax)]. Lymphatic irradiation (Yes/No). Diabetes history: Present/Absent. Economic status: Middle-high income (Annual income greater than $10,000)/Low income (Annual income less than $10,000), Educational levels: high (college education or above)/low (high school education or below), Treatment modalities: Endocrine therapy (Yes/No), Targeted therapy (Yes/No), Prior chemotherapy (Yes/No). In this study, two methods are generally used for postoperative radiotherapy in breast - conserving and mastectomy patients, namely hypofractionated radiotherapy (43.5Gy/15 fractions) and conventional fractionated radiotherapy (50Gy/25 fractions). Therefore, we changed the CTV dose to a binary categorical variable for representation (<50Gy vs ≥50Gy). All radiotherapy-related target volume definitions and scopes refer to the breast cancer guidelines of the National Comprehensive Cancer Network (NCCN) (6).

The conventional prescription dose for postoperative radiotherapy in breast cancer has been widely recognized globally. According to the consensus guidelines of ESTRO and RTOG as well as numerous clinical studies: Tumor bed boost (Boost): For high-risk patients, the boost dose to the surgical tumor bed area is usually 10–16 Gy, which is administered after completing whole breast/chest wall irradiation (50 Gy/25 fractions). The standard basic prescription dose for the whole breast/chest wall (CTVcw/CTVbc) is 50 Gy in 25 fractions. This dose has been proven to effectively control subclinical lesions, with clear efficacy and acceptable toxicity. Although the single-fraction dose and total dose may vary among different patients (such as the use of hypofractionated regimens), their biological effects are usually equivalently converted (EQD2) to the level of 50 Gy. Therefore, 50 Gy is used as the grouping threshold.

Trained radiation oncologists assessed RD severity weekly during radiotherapy and for two weeks post-treatment using the RTOG Acute Radiation Morbidity Scoring Criteria. Discrepancies were resolved through adjudication by the principal investigator. Given the self-limiting nature of Grade 1 dermatitis versus the clinical significance of severe RD (≥Grade 2), the primary endpoint was defined as peak RD severity within this observation window. Cases were dichotomized: severe RD (Grade ≥2, coded 1, N = 364) vs. mild reactions (Grade <2, coded 0, N = 327) (**Supplementary Materials Table S1**).

### 2.3 RTOG acute radiation morbidity scoring criteria

0: No change from baseline
1: Follicular/mild erythema; dry desquamation; decreased sweating
2: Tender/bright erythema; patchy moist desquamation; moderate edema
3: Confluent moist desquamation (excluding skin folds); pitting edema
4: Ulceration, hemorrhage, or necrosis

### 2.4 Data preprocessing

Data preprocessing was performed using the Pandas (Python Data Analysis Library) for cleaning, categorical variable encoding, and standardization. The library/versioning information for the various packages/software used, the code hosted on GitHub and the TRIPOD checklists were in the supplementary materials. The dataset was random split stratified into training (80%) and test (20%) cohorts.

### 2.5 Statistical analysis

All analyses were conducted using Python 3.9 and R 4.2. Quantitative variables (age, height, weight, BMI, body surface area) were expressed as mean ± standard deviation and compared via independent t-tests. Categorical variables (education level, diabetes status, socioeconomic factors, surgical/treatment modalities, tumor characteristics) were presented as frequencies (%) with chi-square testing. Statistical significance was determined at *p* < 0.05. The grid search was used for hyperparameter optimization.

### 2.6 Model development and validation

The predictive performance of fourteen machine learning algorithms was systematically evaluated using training data. Model training incorporated 10-fold cross-validation to maximize generalizability. This technique iteratively partitioned data into 10 equal subsets, with each subset serving as the test cohort once while the remaining nine subsets trained the model. This rotational test protocol ensured comprehensive utilization of all data points for both training and testing phases, thereby improving performance estimation reliability. Hyperparameter optimization was conducted via grid search. Although we did not perform feature selection, we ultimately assessed the importance of each feature and the impact of feature interventions on the final predictive outcomes.

**Figure 1:**
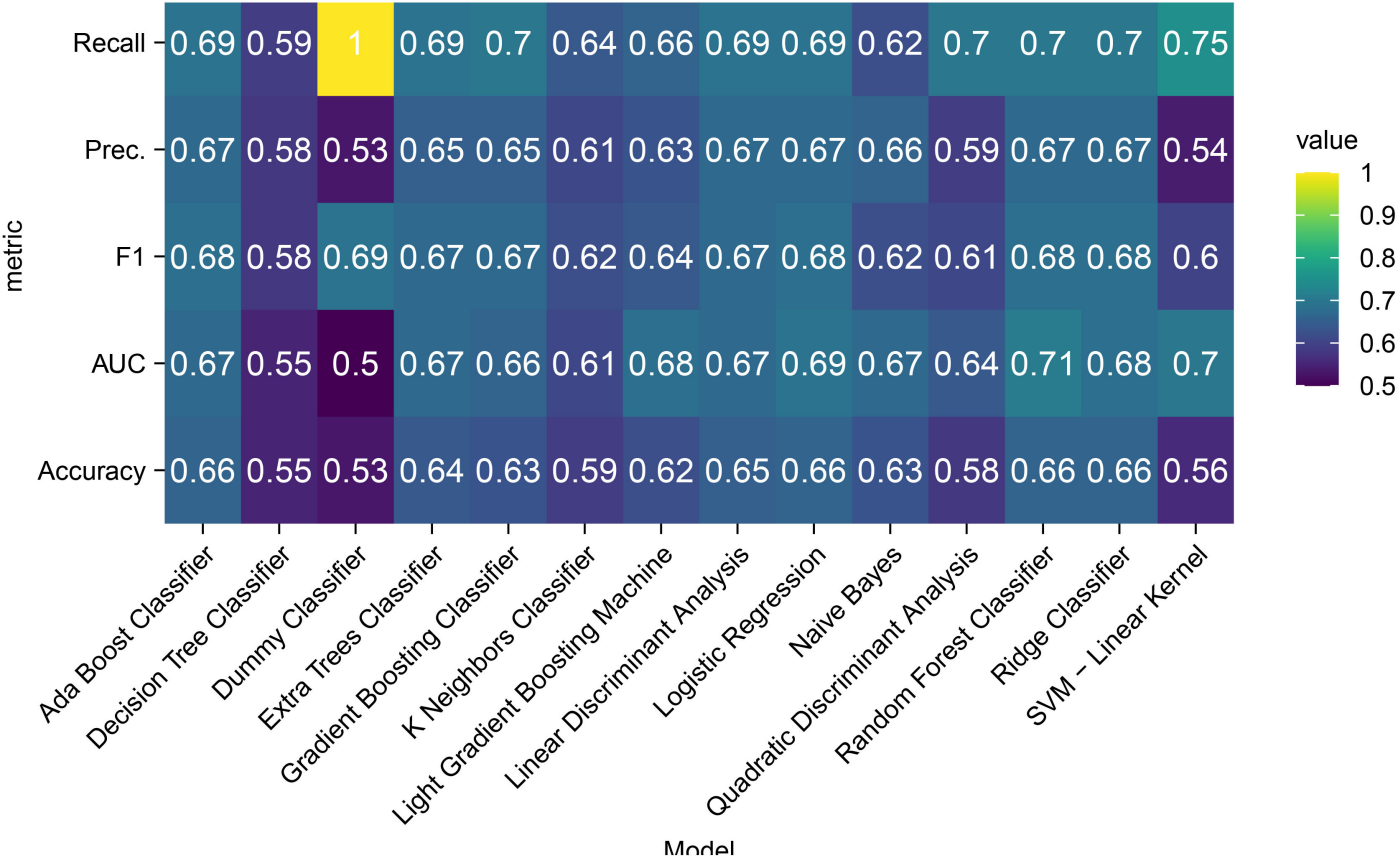
Heatmap of classification performance metrics for different models.

A comparative heatmap (**Figure 1**) visualized key metrics across test cohorts: recall, precision, F1-score, AUC-ROC, and accuracy. The random forest (RF) classifier demonstrated optimal balanced performance and was selected for final model construction.

The discriminative capacity of random forest model was primarily assessed using the AUC. ROC curves illustrate the relationship between true positive rate (sensitivity) and false positive rate (1-specificity), representing classifier performance across all decision thresholds. An AUC of 0.5 indicates random prediction, while 1.0 denotes perfect discrimination. Calibration was evaluated via calibration curves. Decision Curve Analysis (DCA) quantified clinical utility by calculating net benefit across treatment thresholds to assess real-world clinical value.

### 2.7 Model interpretation

SHAP values were employed for predictive interpretation, leveraging cooperative game theory principles to equitably allocate feature contributions. This approach quantified individual feature impacts on predictions and identified critical determinants of RD risk. The methodology not only validated diagnostic accuracy but also elucidated pathophysiological relationships between predictors and outcomes, thereby enhancing clinical model reliability.

## 3 Results

### 3.1 Key findings

We successfully developed and validated a multidimensional clinical prediction model for acute radiation dermatitis, identifying radiotherapy target volumes (CTVsc, CTVim), TNM stage, and diabetic status as pivotal predictors. SHAP interpretability analysis quantified nonlinear feature interactions within the model.

### 3.2 Benchmark testing

Comparative evaluation of 14 machine learning algorithms (**Figure 1**) revealed distinct performance patterns across evaluation metrics. Notably, precision parity was observed among five algorithms—random forest, AdaBoost, linear discriminant analysis, logistic regression, and ridge classifiers—all achieving equivalent precision scores (0.67). While the dummy classifier demonstrated superior F1-score performance (0.69), this apparent advantage likely stems from dataset-specific class distribution biases rather than genuine predictive capability. The random forest model securing the highest AUC (0.71) while sharing peak accuracy (0.66) with AdaBoost, logistic regression, and ridge classifiers. The random forest’s balanced performance profile, particularly its AUC superiority and shared accuracy leadership, supports its selection as the optimal compromise between discriminative power and clinical interpretability.

### 3.3 Model performance evaluation

#### 3.3.1 Discriminative capacity

**Figure 2:**
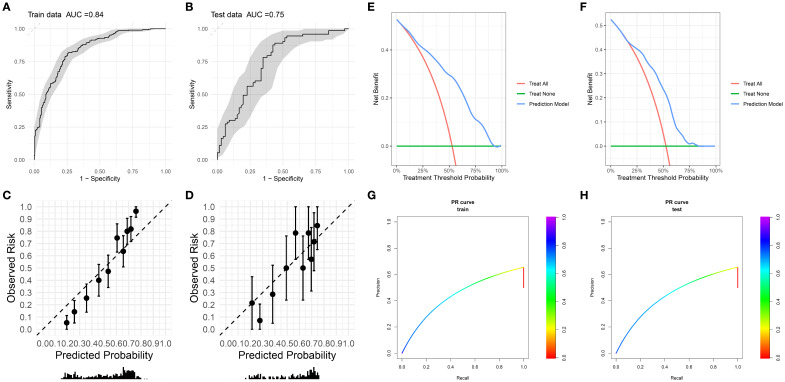
**(A, B)** Receiver operating characteristic (ROC) curves with corresponding area under the curve (AUC) for training and testing datasets. **(C, D)** Calibration plots of observed risk versus predicted probability for training dataset and testing dataset. **(E, F)** Decision curve analysis of net benefit for different treatment strategies and prediction model across treatment threshold probabilities. **(G, H)** Precision - recall (PR) curves for training dataset and testing dataset.

The random forest model exhibited robust performance across cohorts (**Figures 2A, B**): Training set (n=552): AUC 0.84 (95% CI:0.807-0.873), sensitivity 0.811 (76.6%-85.6%), specificity 0.747 (69.4%-80.0%). test set (n=139): AUC 0.748 (95% CI:0.665-0.831), sensitivity 0.877 (80.1%-95.2%), specificity 0.576 (45.7%-69.5%).

#### 3.3.2 Calibration performance

The calibration curve was used to evaluate the degree of agreement between the predicted probabilities of the model and the actually observed risks. The calibration curves of the training set (**Figure 2A**) and the test set (**Figure 2B**) showed that the overall degree of agreement between the predicted probabilities (Predicted Probability) and the observed risks (Observed Risk) was good. The distribution trend of the data points along the diagonal line (dashed line) was relatively regular, suggesting that the model had a certain reliability in predicting risks within both the training set and the test set, demonstrating basic risk prediction calibration characteristics, and laying a foundation for further optimization and clinical application.

The calibration slope of the training set is 1.86 (95% CI: 1.55-2.77), the calibration intercept is 0.03 (95% CI: -0.15-0.21), and the Brier score is 0.173; the calibration slope of the internal holdout test set is 1.35 (95% CI: 0.82-1.89), the calibration intercept is -0.06 (95% CI: -0.42-0.30), and the Brier score is 0.198.

#### 3.3.3 Clinical Utility

Decision curve analysis (DCA, **Figures 2E, F**) revealed superior net benefit (0.2-0.4) versus “treat-all” and “treat-none” strategies across therapeutic thresholds (25%-75%). Model efficacy diminished at extreme thresholds (<25% or >75%) but remained preferable to nontreatment. This supports risk-stratified clinical decision-making, minimizing overtreatment in low-risk patients (threshold >50%) and undertreatment in high-risk subgroups (threshold <25%).

### 3.4 SHAP feature importance analysis

**Figure 3:**
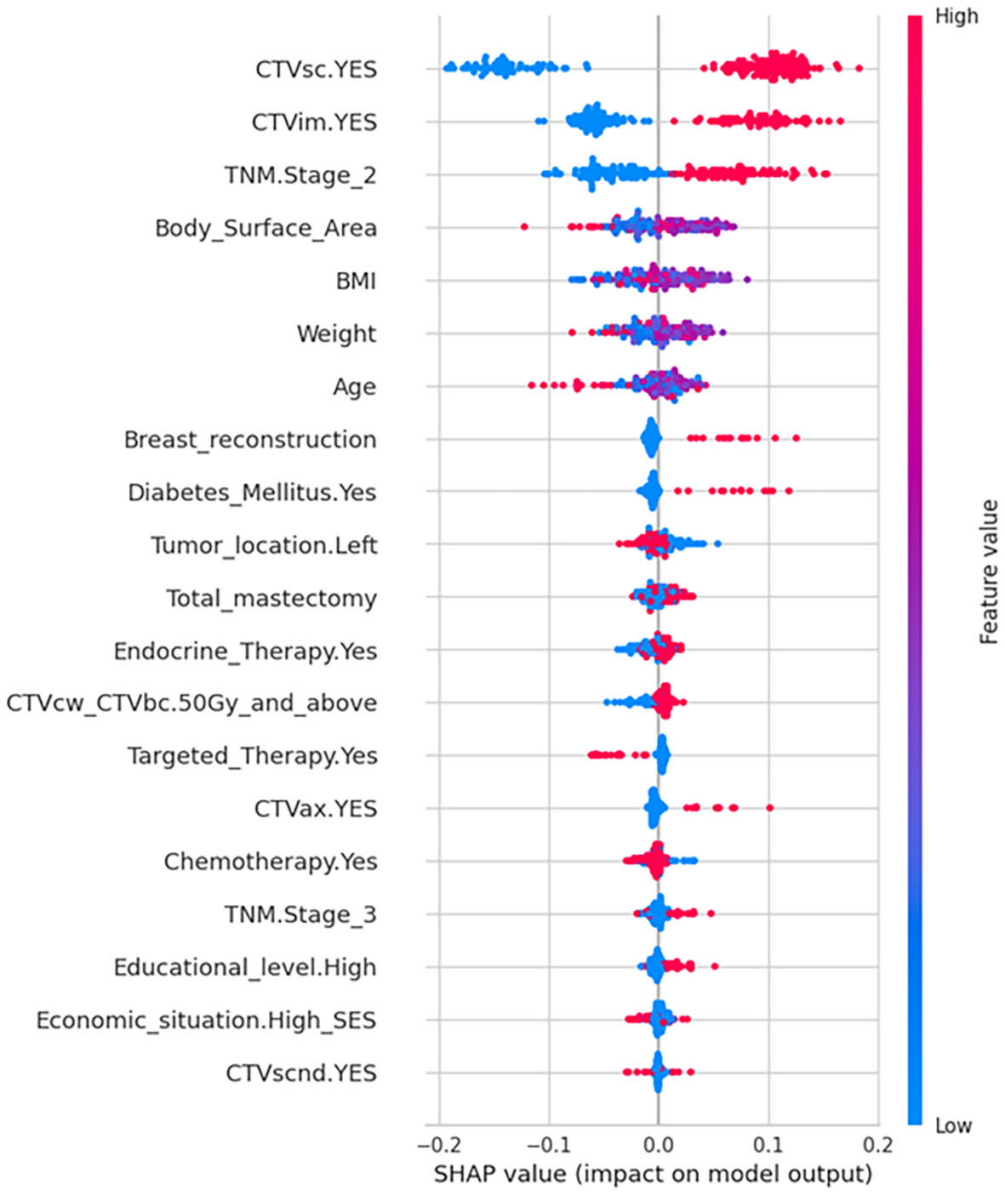
SHAP summary plot of feature importance for model interpretation. SHAP directionality plot illustrating the value of predictors to the output of random forest model. Higher relative feature values are shaded in red and lower values in blue for each variable. The SHAP value on the x-axis represents the influence of each data point on the model output, with positive values contributing to a positive prediction (higher risk of Grade 2/3 RD), and negative values contributing towards a negative prediction (lower risk of Grade 2/3 RD). Binary variables (CTVsc, CTVim, Diabetes, Endocrine Therapy, Targeted Therapy, CTVax, Chemotherapy, Economic situation, CTVscnd) were dichotomized as 1= Yes, 0=No.

**Figure 3** displays the SHAP summary plot, illustrating global feature importance in the random forest model for predicting grade ≥2 RD risk in breast cancer. Key insights emerge:1. Feature Hierarchy: Top-ranked features (CTVsc, CTVim, TNM Stage 2) exhibit the strongest predictive influence, with wider dispersion of SHAP values indicating greater contribution variability. 2.Value-Outcome Correlation: Red dots (high feature values) cluster on the plot’s right (positive SHAP values → increased RD risk), while blue dots (low values) dominate the left (negative SHAP values → risk reduction).3. Clinical-Mechanistic Consistency: Broad radiotherapy targets (CTVsc/CTVim) align with dose-dependent skin toxicity mechanisms. CTVcwCTVbc ≥50 Gy and TNM Stage 2 show dose-response relationships consistent with clinical observations. Notably, breast reconstruction, diabetic status, and Clinical Target Volume-Axilla (CTVax) demonstrate right-skewed distributions, suggesting high values disproportionately elevate risk. Conversely, targeted therapy exhibits left-skewed distribution, implying protective effects against severe RD.

### 3.5 Morris sensitivity analysis

**Figure 4:**
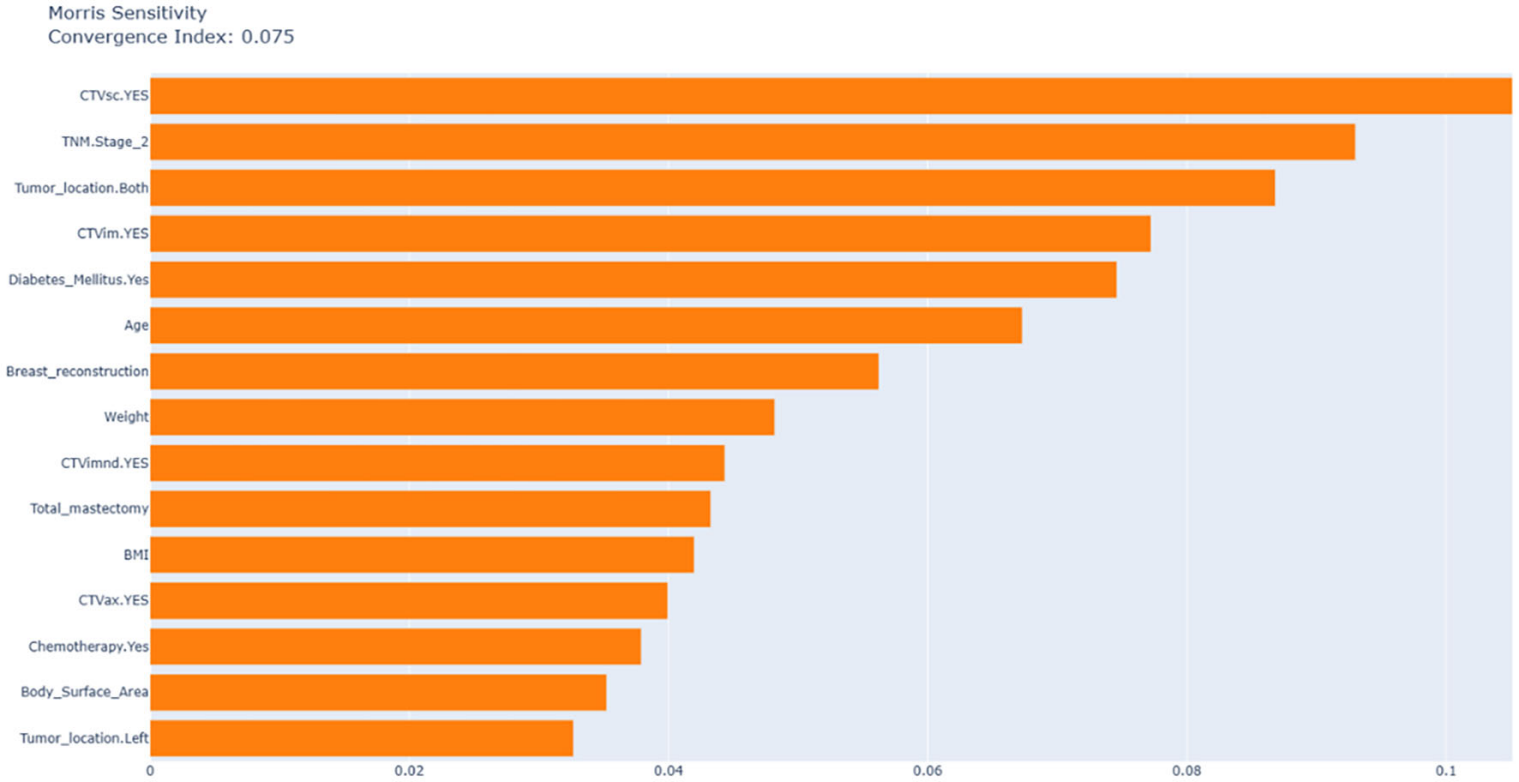
Morris sensitivity analysis of feature importance with convergence index.

**Figure 4** uses Morris analysis to quantify the global feature sensitivity with a convergence index of 0.075 confirming the stability of the results. The key findings are as follows: First, in terms of dominant predictors, supraclavicular CTVsc (sensitivity index: 0.105), CTVim (0.077), and TNM Stage 2 (0.093) are the main factors driving model variability, which is consistent with the results of SHAP analysis. Second, diabetic status exhibits a high SHAP contribution (approximately 0.08) but low Morris sensitivity.

### 3.6 Individualized risk stratification

**Figure 5:**
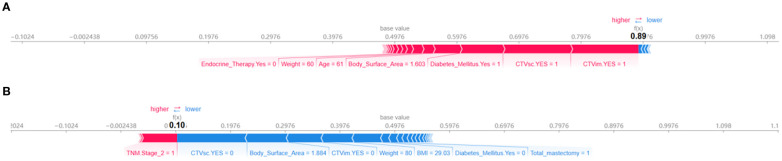
SHAP force plots for interpreting model predictions on individual instances. **(A)** Positive force-directed graph. **(B)** Negative force-directed graph.

**Figure 5** employs SHAP force plots to illustrate patient-specific risk drivers. Positive drivers (factors increasing the predicted risk) include prophylactic internal mammary/supraclavicular radiotherapy, TNM Stage 2, diabetes, and a low body surface area (1.603 m²). In contrast, negative drivers (factors reducing the predicted risk) involve the omission of internal mammary/supraclavicular radiotherapy. This visualization systematically links individual clinical characteristics and treatment decisions to personalized risk assessments, offering a clear interpretation of how each factor contributes to the model’s predictions.

## 4 Discussion

Given the paucity of predictive models integrating radiotherapy target volumes and dosimetric parameters for breast cancer radiation dermatitis, this study addresses acute radiation dermatitis—a clinically significant and prevalent complication in breast cancer management—by developing a machine learning-based predictive framework. The model integrates multi-dimensional clinical, treatment-related, and socioeconomic variables, leveraging ensemble learning algorithms coupled with SHAP-based interpretability analysis. Key risk factors identified through this approach include radiotherapy target volumes (CTVsc, CTVim), TNM Stage II, and diabetic status, underscoring the utility of comprehensive feature integration for improving risk stratification in clinical practice.

We selected 1–2 methods from each category of machine learning models, compiling a total of 14 machine learning models, which represent all current mainstream machine learning approaches. Through heatmaps and combined with five metrics including accuracy and recall rate, the random forest model with the most balanced performance was selected to construct the prediction model. This approach is more scientific and yields higher accuracy and credibility compared to previous studies that used a single machine learning method to build prediction models. Compared to other machine learning models, the random forest model offers several key advantages. Its ensemble nature, leveraging multiple decision trees with voting or averaging, effectively mitigates overfitting by reducing variance, enabling reliable performance on complex datasets. The model’s proficiency in handling high-dimensional and noisy data is notable; it automatically identifies and ranks feature importance, filtering out irrelevant variables efficiently. Parallel training of decision trees significantly boosts computational efficiency, particularly beneficial for large-scale datasets. Moreover, random forest demonstrates robust performance regardless of outliers or missing values, and its applicability spans classification and regression tasks without stringent assumptions on data distribution. Its simplicity and interpretable feature importance metrics further enhance accessibility, bridging the gap between technical and non-technical stakeholders (7).

According to clinical practice, most patients who have undergone breast - conserving surgery and mastectomy need radiotherapy. Therefore, in order to ensure the maximum scope of application of the model, we included patients who had breast - conserving surgery and total mastectomy. The study found that the impact of these two surgical approaches on radiation dermatitis was not significant, which also validates the rationality of including these two categories of patients. Among 691 breast cancer patients, the incidence of grade ≥2 acute radiation dermatitis was 52.68%, consistent with findings from high-quality meta-analyses (8). SHAP algorithm identified radiation field, TNM stage, and diabetes as the most significant predictors of severe radiation dermatitis. It is worth noting that although the SHAP analysis focuses on the best - performing random forest model to deeply explain its prediction mechanism, to ensure the robustness of the key findings, we also conducted a Morris analysis on the model (**Figure 4**). CTVsc (sensitivity index: 0.105), CTVim (0.077), and TNM Stage 2 (0.093) are the main factors driving model variability, which is consistent with the results of the SHAP analysis. Analysis of baseline characteristics between the training cohort (N = 552) and test cohort (N = 139) revealed disparities in tumor location and pathological subtypes. In the training cohort, left-breast tumors accounted for 54.0% versus 44.7% right-breast tumors, whereas the test cohort showed 41.7% left-breast and 56.8% right-breast tumors (P = 0.025). Pathologically, DCIS prevalence was 1.27% in the training cohort versus 3.60% in the test cohort, while LCIS occurred in 2.36% of the training cohort but was absent in the test cohort (P = 0.054). Despite these significant intergroup differences in tumor location and pathological distribution, our predictive model demonstrated robust concordance across both cohorts, indicating strong generalizability and reliability of the findings.

The most direct evidence for evaluating the generalization of the model comes from its performance on a completely independent test set. As shown in **Figure 2**, the final model of this study achieved an AUC of 0.75 on the independent test set, which is highly close to the AUC of 0.84 on the training set, with a performance decay of only 5.29%. This excellent and stable performance on unseen data strongly supports that the model has good generalization ability and is expected to be applicable to the prediction of similar populations in the future.

There is a notable drop in specificity from the training set (0.747) to the test set (0.576). While the validation specificity of 0.576 indicates room for improvement (especially in reducing false positives), we emphasize that this performance characteristic stems from a deliberate choice to prioritize sensitivity to maximize the detection of patients at high risk of radiation dermatitis - which is the main clinical goal of this prediction model. The model exhibits strong overall discriminative ability (AUC = 0.748 (95% CI: 0.665 - 0.831)) and achieves high sensitivity (0.877 (80.1% - 95.2%)), making it a valuable tool for identifying patients most likely to benefit from proactive intervention. We are committed to optimizing the specificity of the model in future iterations through the above - mentioned strategies.

CTVsc and CTVim exhibited significantly higher SHAP contribution values than other features. Their positive status (irradiation presence) resulted in positive SHAP value shifts, identifying them as core drivers of RD risk. CTVsc and CTVim reflect radiotherapy target coverage, directly determining the radiation-exposed skin area. Generally, larger radiation fields correlate with higher probabilities of dermatitis development (9). Furthermore, the supra/infraclavicular and internal mammary lymph node regions, adjacent to the chest wall dermis, often encompass skin folds at the cervicothoracic junction (notable for dose build-up effects). When CTVsc/CTVim coverage is required, multi-field tangential irradiation is typically employed, leading to overlapping skin exposure volumes. This increases cumulative basal layer doses, potentially elevating risks of basal stem cell depletion. It is worth noting that the SHAP analysis shows that the feature sensitivity of tumor location is relatively low. This may reflect that modern precision radiotherapy techniques (such as IMRT/VMAT) can effectively optimize the dose distribution and reduce the direct impact of the tumor location itself on the dose differences in adjacent normal tissues (such as the skin). The model relies more on factors that directly reflect tissue dose (such as target volume, dose parameters) and patient intrinsic factors (such as diabetes status, TNM stage), which is clinically reasonable.

Compared to stage I and earlier patients, TNM stages II and III also influence severe radiation dermatitis incidence, potentially linked to extended surgical margins, intensive chemotherapy regimens, and higher radiation doses in locally advanced cases (10). Notably, TNM stage II exhibited more pronounced effects than stage III, possibly attributable to its larger sample size (n=354) enhancing statistical power, whereas stage III’s limited cohort (n=122) may have constrained effect magnitude detection.

In diabetic patients, chronic hyperglycemia elevates reactive oxygen species (ROS) levels, synergistically amplified by radiotherapy-induced ROS generation, exacerbating cutaneous DNA damage and lipid peroxidation (11, 12). Diabetes-associated microangiopathy reduces dermal perfusion and tissue oxygenation, impairing radiation injury repair. Concurrently, decreased stratum corneum hydration factors and compromised epidermal barrier function heighten radiation sensitivity. These pathophysiological interactions collectively elevate RD risk in diabetic populations.

Beyond primary findings, exploratory analyses revealed intriguing patterns. SHAP summary plots demonstrated right-tailed distributions for features like breast reconstruction and CTVax, indicating heterogeneous positive predictive contributions arising from nonlinear exposure-response relationships, class imbalances, and asymmetric clinical mechanisms. These variables may act as effect modifiers that potentiate other risk factors. Post-mastectomy reconstruction patients showed elevated RD susceptibility, likely due to compounded tissue trauma from implant procedures compared to simple mastectomy (13, 14), which diminishes skin radiation tolerance. Reconstructed breasts typically require larger irradiated skin areas, while compromised vascularization and impaired thermal dissipation near implants further exacerbate injury risks (15, 16). CTVax expansion, particularly in friction-prone regions like axillary skin folds, increases radiation-exposed epithelial vulnerability, though its impact remains secondary to CTVsc/CTVim due to limited axillary irradiation cases (n=52).

As the most critical and extensive target volume, chest wall/whole breast irradiation doses (CTVcw_CTVbc) demonstrated increased RD risk (right-skewed SHAP values) at doses ≥50 Gy, aligning with the established mechanism of dermal microvascular damage from high-dose hotspots. Hypofractionated radiotherapy reduces ARD risk in breast cancer patients compared to conventional fractionation, a finding corroborated by a meta-analysis of 38 studies involving 15,623 patients (17, 18). Anna Zigogianni’s investigation of stage I-II invasive breast cancer patients post-lumpectomy further validated hypofractionation’s protective effect against severe RD (19), suggesting that while conventional fractionation elevates RD risk, its impact remains secondary to radiation field parameters.

Although BMI has been reported as a prognostic factor for RD due to its correlation with breast volume – where higher BMI often corresponds to larger breasts and inframammary fold tension (20–23) – our findings contradict this association. Neither BMI, height, nor weight emerged as significant RD predictors in this study. Both cohorts exhibited mean BMI values exceeding 25 (training: 25.5; test: 25.8), indicating substantial overweight/obesity prevalence. Subgroup analysis revealed comparable RD incidence between BMI <25 (n=316) and BMI ≥25 (n=375) patients. These discrepancies highlight ongoing controversies regarding BMI’s predictive utility for RD, warranting further investigation to clarify its pathophysiological role.

These factors do not operate in isolation but interact synergistically to influence the pathogenesis and progression of radiation dermatitis. Clinicians should adopt integrated risk assessments, particularly prioritizing interventions for patients with multiple high-risk profiles. When the prediction model indicates a high risk, it means that the patient has a relatively high risk of developing radiation dermatitis of grade II or above. In this case, we will consider giving skin protectants in advance rather than waiting until the radiation dermatitis worsens, and instruct the patient to keep the skin in the radiotherapy area clean. Optimize the radiotherapy plan while ensuring the radiotherapy dose. If the patient has diabetes, we will focus on monitoring and adjusting the patient’s blood sugar level, and increase the frequency of observing the patient’s skin condition every week. Given the excellent predictive performance of the model, in the future, we plan to embed it into radiotherapy planning software to support real-time adjustments to the treatment plan.

This study has several limitations. First, the moderate sample size may constrain statistical power for detecting subtle associations. Second, the single-center design restricts generalizability, as institutional-specific protocols and population characteristics may differ across regions. Third, the information on pathological type was not included as a predictor in this study, mainly due to the high missing rate of this variable in the retrospective data. Although certain pathological types may be associated with the radiotherapy response, this model has demonstrated good predictive performance (AUC = 0.75) on the internal hold-out test set without using this information. Future prospective studies should systematically collect pathological data to further optimize the model. Despite these constraints, our findings provide foundational insights for future research. Through this preliminary study, we have identified the most influential variables (CTVsc, CTVim, TNM stage, and diabetic status). In the next step, we will develop and validate new models based on these factors in a larger population.

## Data Availability

The raw data supporting the conclusions of this article will be made available by the authors, without undue reservation.
