# Supplementary material for: Development and validation of an interpretable machine learning model for acute radiation dermatitis in breast cancer
Source: Front Oncol. 2025 Oct 17;15:1663293. doi: 10.3389/fonc.2025.1663293 (PMC12575144; doi:10.3389/fonc.2025.1663293)
Supplement: Supplementary file 1 [file DataSheet1.pdf]

Abbreviation：

Radiation dermatitis (RD)
Radiation Therapy Oncology Group (RTOG)
Area Under the Curve (AUC)
Shapley Additive exPlanations (SHAP)
Clinical Target Volume-Supraclavicular (CTVsc)
Clinical Target Volume-Internal Mammary (CTVim)
acute radiation dermatitis (ARD)
Image-Guided Intensity-Modulated Radiation Therapy (IGRT-IMRT)
Ductal Carcinoma In Situ (DCIS)
Lobular Carcinoma In Situ (LCIS)
Invasive breast carcinoma (IBC)
Clinical Target Volume-Chest Wall (CTVcw)
Clinical Target Volume-Breast Cancer (CTVbc)
The random forest (RF)
Decision Curve Analysis (DCA)
Clinical Target Volume-Axilla (CTVax)

Supplementary Materials-table S1

Comparison of Baseline Characteristics and Clinical Variables between the Training Group and the Test Group of Radiotherapy Patients

	**modeling**	**test**	**p.overall**
	N=552	***N=139***	
ID	344 (199)	353 (202)	0.639
Age	51.8 (10.7)	52.0 (11.7)	0.841
Educational_level:			1.000
High	137 (24.8%)	34 (24.5%)	
Low	415 (75.2%)	105 (75.5%)	
Height	1.66 (0.86)	1.59 (0.05)	0.089
Weight	65.5 (10.5)	65.7 (10.8)	0.861
BMI	25.5 (4.14)	25.8 (3.97)	0.365
Body_Surface_Area	1.70 (0.54)	1.66 (0.15)	0.175
Diabetes_Mellitus:			0.190
No	510 (92.4%)	123 (88.5%)	
Yes	42 (7.61%)	16 (11.5%)	
Economic_situation:			0.226
High_SES	107 (19.4%)	34 (24.5%)	
Low_SES	445 (80.6%)	105 (75.5%)	
Surgical_approach:			0.923
Breast_conserving	279 (50.5%)	68 (48.9%)	
Breast_reconstruction	49 (8.88%)	12 (8.63%)	
Total_mastectomy	224 (40.6%)	59 (42.4%)	
Endocrine_Therapy:			0.191
No	218 (39.5%)	64 (46.0%)	
Yes	334 (60.5%)	75 (54.0%)	
Targeted_Therapy:			0.467
No	520 (94.2%)	128 (92.1%)	

	modeling	test	p.overall
	N=552	*N=139*	
Yes	32 (5.80%)	11 (7.91%)	
Chemotherapy:			0.455
No	105 (19.0%)	22 (15.8%)	
Yes	447 (81.0%)	117 (84.2%)	
Tumor_location:			0.025
Both	7 (1.27%)	2 (1.44%)	
Left	298 (54.0%)	58 (41.7%)	
Right	247 (44.7%)	79 (56.8%)	
Pathology:			0.054
DCIS	7 (1.27%)	5 (3.60%)	
IBC	523 (94.7%)	131 (94.2%)	
LCIS	13 (2.36%)	0 (0.00%)	
other	9 (1.63%)	3 (2.16%)	
TNM:			0.578
Stage_0_to_1	194 (35.1%)	45 (32.4%)	
Stage_2	254 (46.0%)	62 (44.6%)	
Stage_3	92 (16.7%)	30 (21.6%)	
Stage_4	12 (2.17%)	2 (1.44%)	
CTVcw_CTVbc:			0.685
50Gy_and_above	393 (71.2%)	102 (73.4%)	
Below_50Gy	159 (28.8%)	37 (26.6%)	
CTVsc:			0.503
NO	202 (36.6%)	46 (33.1%)	
YES	350 (63.4%)	93 (66.9%)	
CTVscnd:			0.694
NO	516 (93.5%)	128 (92.1%)	

	modeling	test	p.overall
	N=552	***N=139***	
YES	36 (6.52%)	11 (7.91%)	
CTVim:			0.253
NO	322 (58.3%)	73 (52.5%)	
YES	230 (41.7%)	66 (47.5%)	
CTVimnd:			0.794
NO	534 (96.7%)	134 (96.4%)	
YES	18 (3.26%)	5 (3.60%)	
CTVax:			0.708
NO	512 (92.8%)	127 (91.4%)	
YES	40 (7.25%)	12 (8.63%)	
Radiodermatitis	1.59 (0.61)	1.65 (0.70)	0.352

P value <0.05. Abbreviations =TNM, TNM staging; CTVcw, Clinical Target Volume - Chest Wall; CTVbc, Clinical Target Volume - Breast Cancer; CTVsc, Clinical Target Volume - Supraclavicular; CTVscnd, Clinical Target Volume - Supraclavicular Nodes; CTVim, Clinical Target Volume - Internal Mammary; CTVimnd, Clinical Target Volume - Internal Mammary Node; CTVax, Clinical Target Volume – Axilla

GitHub：

https://github.com/zhangkaicr/DVIM-ARDBC

Supplementary Materials-table s2 roc_train

AUC	AUC.SE	AUC.low	AUC.up	P	ACC
0.840	0.017	0.807	0.873	0.000	0.781
ACC.low	ACC.up	SEN	SEN.low	SEN.up	SPE
0.780	0.781	0.811	0.766	0.856	0.747
SPE.low	SPE.up	PLR	PLR.low	PLR.up	NLR
0.694	0.800	3.207	2.585	3.980	0.253
NLR.low	NLR.up	PPV	PPV.low	PPV.up	NPV
0.197	0.324	0.781	0.735	0.828	0.780
NPV.low	NPV.up	PPA	PPA.low	PPA.up	NPA
0.729	0.831	0.811	0.766	0.856	0.747
NPA.low	NPA.up	TPA	TPA.low	TPA.up	KAPPA
0.694	0.800	0.781	0.746	0.815	0.559
KAPPA.low	KAPPA.up				

Supplementary Materials-table s3 roc test

AUC	AUC.SE	AUC.low	AUC.up	P	ACC
0.748	0.042	0.665	0.831	0.000	0.734
ACC.low	ACC.up	SEN	SEN.low	SEN.up	SPE
0.731	0.737	0.877	0.801	0.952	0.576
SPE.low	SPE.up	PLR	PLR.low	PLR.up	NLR
0.457	0.695	2.067	1.540	2.773	0.214
NLR.low	NLR.up	PPV	PPV.low	PPV.up	NPV
0.112	0.408	0.696	0.602	0.790	0.809
NPV.low	NPV.up	PPA	PPA.low	PPA.up	NPA
0.696	0.921	0.877	0.801	0.952	0.576
NPA.low	NPA.up	TPA	TPA.low	TPA.up	KAPPA
0.457	0.695	0.734	0.660	0.807	0.459
KAPPA.low	KAPPA.up				
0.316	0.602				

```
sys: 3.10.8 (main, Nov 24 2022, 14:13:03) [GCC 11.2.0]
marshal: 4
re: 2.2.1
ipykernel._version: 6.20.2
json: 2.0.9
jupyter_client._version: 8.0.1
logging: 0.5.1.2
platform: 1.0.8
_ctypes: 1.1.0
ctypes: 1.1.0
zmq.sugar.version: 25.0.0
zmq.sugar: 25.0.0
zmq: 25.0.0
traitlets._version: 5.8.1
traitlets: 5.8.1
jupyter_core.version: 5.1.5
jupyter_core: 5.1.5
tornado: 6.2
zlib: 1.0
_curses: b'2.2'
curses: b'2.2'
socketserver: 0.4
argparse: 1.1
dateutil._version: 2.8.2
dateutil: 2.8.2
six: 1.16.0
_decimal: 1.70
decimal: 1.70
platformdirs.version: 2.6.2
platformdirs: 2.6.2
_csv: 1.0
csv: 1.0
importlib.metadata: <function version at 0x7fa518e3b2e0>
jupyter_client: 8.0.1
ipykernel: 6.20.2
IPython.core.release: 8.9.0
executing.version: 1.2.0
executing: 1.2.0
pure_eval.version: 0.2.2
pure_eval: 0.2.2
stack_data.version: 0.6.2
stack_data: 0.6.2
pygments: 2.14.0
ptyprocess: 0.7.0
pexpect: 4.8.0
IPython.core.crashhandler: 8.9.0
pickleshare: 0.7.5
backcall: 0.2.0
decorator: 5.1.1
_sqlite3: 2.6.0
sqlite3.dbapi2: 2.6.0
sqlite3: 2.6.0
wcwidth: 0.2.6
prompt_toolkit: 3.0.36
```

```
parso: 0.8.3
jedi: 0.18.2
urllib.request: 3.10
IPython.core.magics.code: 8.9.0
IPython: 8.9.0
comm: 0.1.2
psutil._psutil_linux: 594
psutil: 5.9.4
debugpy.public_api: 1.6.6
debugpy: 1.6.6
xmlrpc.client: 3.10
http.server: 0.6
pkg_resources._vendor.more_itertools: 8.12.0
pkg_resources.extern.more_itertools: 8.12.0
pkg_resources._vendor.appdirs: 1.4.3
pkg_resources.extern.appdirs: 1.4.3
pkg_resources._vendor.packaging.__about__: 21.3
pkg_resources._vendor.packaging: 21.3
pkg_resources.extern.packaging: 21.3
pkg_resources._vendor.pyparsing: 3.0.9
pkg_resources.extern.pyparsing: 3.0.9
_pydevd_frame_eval.vendored.bytecode: 0.13.0.dev
_pydevd_bundle.pydevd_cython: 11
_pydevd_bundle.pydevd_cython_wrapper: 11
_pydevd_bundle.pydevd_additional_thread_info_regular: 11
_pydev_bundle.fsnotify: 0.1.5
pydevd: 2.9.5
packaging: 23.0
pycaret.utils: 3.3.2
setuptools._distutils: 3.10.8
setuptools.version: 65.5.0
setuptools._vendor.packaging.__about__: 21.3
setuptools._vendor.packaging: 21.3
setuptools.extern.packaging: 21.3
setuptools._vendor.ordered_set: 3.1
setuptools.extern.ordered_set: 3.1
setuptools._vendor.more_itertools: 8.8.0
setuptools.extern.more_itertools: 8.8.0
setuptools._vendor.pyparsing: 3.0.9
setuptools.extern.pyparsing: 3.0.9
setuptools.config.expand: <function version at 0x7fa5057d0310>
setuptools.dist: <module 'setuptools.extern.packaging.version' from '/root/miniconda3/lib/
setuptools.depends: <module 'setuptools.extern.packaging.version' from '/root/miniconda3/l
setuptools: 65.5.0
distutils: 3.10.8
importlib_metadata: <function version at 0x7fa5056aeb00>
wurlitzer: 3.1.1
pycaret: 3.3.2
numpy.version: 1.24.1
numpy.core._multiarray_umath: 3.1
numpy.core: 1.24.1
numpy.linalg._umath_linalg: 0.1.5
numpy.lib: 1.24.1
numpy: 1.24.1
```

```
scipy.version: 1.11.4
scipy: 1.11.4
scipy.sparse.linalg._isolve._iterative: 1.21.6
scipy._lib.decorator: 4.0.5
scipy.linalg._fblas: 1.21.6
scipy.linalg._flapack: 1.21.6
scipy.linalg._flinalg: 1.21.6
scipy.sparse.linalg._eigen.arpack._arpack: 1.21.6
joblib.externals.cloudpickle: 2.2.0
joblib.externals.loky: 3.4.1
joblib: 1.3.2
sklearn.utils._joblib: 1.3.2
scipy.special._specfun: 1.21.6
scipy.optimize._minpack2: 1.21.6
scipy.optimize._lbfgsb: 1.21.6
scipy.optimize._cobyla: 1.21.6
scipy.optimize._slsqp: 1.21.6
scipy.optimize.__nnls: 1.21.6
scipy.linalg._interpolative: 1.21.6
scipy.integrate._vode: 1.21.6
scipy.integrate._dop: 1.21.6
scipy.integrate._lsoda: 1.21.6
scipy.interpolate.dfitpack: 1.21.6
scipy._lib._uarray: 0.8.8.dev0+aa94c5a4.scipy
scipy.stats._statlib: 1.21.6
scipy.stats._mvn: 1.21.6
threadpoolctl: 3.5.0
sklearn.externals._packaging: <module 'sklearn.externals._packaging.version' from '/root/[illegible]
sklearn.utils._estimator_html_repr: 1.4.2
sklearn.base: 1.4.2
sklearn.utils._show_versions: 1.4.2
sklearn: 1.4.2
```

TRIPOD Checklist: Prediction Model Development and Validation

Section/Topic	Item		Checklist Item	Page
**Title and abstract**				
Title	1	D;V	Identify the study as developing and/or validating a multivariable prediction model, the target population, and the outcome to be predicted.	1
Abstract	2	D;V	Provide a summary of objectives, study design, setting, participants, sample size, predictors, outcome, statistical analysis, results, and conclusions.	1
**Introduction**				
Background and objectives	3a	D;V	Explain the medical context (including whether diagnostic or prognostic) and rationale for developing or validating the multivariable prediction model, including references to existing models.	1-2
	3b	D;V	Specify the objectives, including whether the study describes the development or validation of the model or both.	1-2
**Methods**				
Source of data	4a	D;V	Describe the study design or source of data (e.g., randomized trial, cohort, or registry data), separately for the development and validation data sets, if applicable.	2-3
	4b	D;V	Specify the key study dates, including start of accrual; end of accrual; and, if applicable, end of follow-up.	2
Participants	5a	D;V	Specify key elements of the study setting (e.g., primary care, secondary care, general population) including number and location of centres.	2
	5b	D;V	Describe eligibility criteria for participants.	3
	5c	D;V	Give details of treatments received, if relevant.	3
Outcome	6a	D;V	Clearly define the outcome that is predicted by the prediction model, including how and when assessed.	3-4
	6b	D;V	Report any actions to blind assessment of the outcome to be predicted.	3
Predictors	7a	D;V	Clearly define all predictors used in developing or validating the multivariable prediction model, including how and when they were measured.	3-4
	7b	D;V	Report any actions to blind assessment of predictors for the outcome and other predictors.	4
Sample size	8	D;V	Explain how the study size was arrived at.	2
Missing data	9	D;V	Describe how missing data were handled (e.g., complete-case analysis, single imputation, multiple imputation) with details of any imputation method.	2
Statistical analysis methods	10a	D	Describe how predictors were handled in the analyses.	4-5
	10b	D	Specify type of model, all model-building procedures (including any predictor selection), and method for internal validation.	4-5
	10c	V	For validation, describe how the predictions were calculated.	4
	10d	D;V	Specify all measures used to assess model performance and, if relevant, to compare multiple models.	5
	10e	V	Describe any model updating (e.g., recalibration) arising from the validation, if done.	2
Risk groups	11	D;V	Provide details on how risk groups were created, if done.	2
Development vs. validation	12	V	For validation, identify any differences from the development data in setting, eligibility criteria, outcome, and predictors.	2
**Results**				
Participants	13a	D;V	Describe the flow of participants through the study, including the number of participants with and without the outcome and, if applicable, a summary of the follow-up time. A diagram may be helpful.	5
	13b	D;V	Describe the characteristics of the participants (basic demographics, clinical features, available predictors), including the number of participants with missing data for predictors and outcome.	5
	13c	V	For validation, show a comparison with the development data of the distribution of important variables (demographics, predictors and outcome).	6
Model development	14a	D	Specify the number of participants and outcome events in each analysis.	6
	14b	D	If done, report the unadjusted association between each candidate predictor and outcome.	2
Model specification	15a	D	Present the full prediction model to allow predictions for individuals (i.e., all regression coefficients, and model intercept or baseline survival at a given time point).	2
	15b	D	Explain how to the use the prediction model.	7
Model performance	16	D;V	Report performance measures (with CIs) for the prediction model.	6
Model-updating	17	V	If done, report the results from any model updating (i.e., model specification, model performance).	2
**Discussion**				
Limitations	18	D;V	Discuss any limitations of the study (such as nonrepresentative sample, few events per predictor, missing data).	11
Interpretation	19a	V	For validation, discuss the results with reference to performance in the development data, and any other validation data.	8-10
	19b	D;V	Give an overall interpretation of the results, considering objectives, limitations, results from similar studies, and other relevant evidence.	8
Implications	20	D;V	Discuss the potential clinical use of the model and implications for future research.	11
**Other information**				
Supplementary information	21	D;V	Provide information about the availability of supplementary resources, such as study protocol, Web calculator, and data sets.	Yes
Funding	22	D;V	Give the source of funding and the role of the funders for the present study.	yes

*Items relevant only to the development of a prediction model are denoted by D, items relating solely to a validation of a prediction model are denoted by V, and items relating to both are denoted D;V. We recommend using the TRIPOD Checklist in conjunction with the TRIPOD Explanation and Elaboration document.
